# Air pollution induces pyroptosis of human monocytes through activation of inflammasomes and Caspase-3-dependent pathways

**DOI:** 10.1186/s12950-023-00353-y

**Published:** 2023-08-10

**Authors:** Adrianna Gałuszka-Bulaga, Karolina Tkacz, Kazimierz Węglarczyk, Maciej Siedlar, Jarek Baran

**Affiliations:** 1 Department of Clinical Immunology, Institute of Pediatrics, Jagiellonian University Medical College, Wielicka Street 265, 30-663 Krakow, Poland; 2 Department of Clinical Immunology, University Children’s Hospital, Krakow, Poland

**Keywords:** Particulate matter, Peripheral blood monocytes, Pyroptosis, NLRP3 inflammasome activation, Cytokine production

## Abstract

According to the World Health Organization (WHO), air pollution is one of the most serious threats for our planet. Despite a growing public awareness of the harmful effects of air pollution on human health, the specific influence of particulate matter (PM) on human immune cells remains poorly understood. In this study, we investigated the effect of PM on peripheral blood monocytes in vitro. Monocytes from healthy donors (HD) were exposed to two types of PM: NIST (SRM 1648a, standard urban particulate matter from the US National Institute for Standards and Technology) and LAP (SRM 1648a with the organic fraction removed). The exposure to PM-induced mitochondrial ROS production followed by the decrease of mitochondrial membrane potential and activation of apoptotic protease activating factor 1 (Apaf-1), Caspase-9, and Caspase-3, leading to the cleavage of Gasdermin E (GSDME), and initiation of pyroptosis. Further analysis showed a simultaneous PM-dependent activation of inflammasomes, including NLRP3 (nucleotide-binding oligomerization domain-like receptor pyrin domain containing 3) and Caspase-1, followed by cleavage of Gasdermin D (GSDMD) and secretion of IL-1β. These observations suggest that PM-treated monocytes die by pyroptosis activated by two parallel signaling pathways, related to the inorganic and organic PM components. The release of IL-1β and expression of danger-associated molecular patterns (DAMPs) by pyroptotic cells further activated the remnant viable monocytes to produce inflammatory cytokines (TNF-α, IL-6, IL-8) and protected them from death induced by the second challenge with PM.

In summary, our report shows that PM exposure significantly impacts monocyte function and induces their death by pyroptosis. Our observations indicate that the composition of PM plays a crucial role in this process—the inorganic fraction of PM is responsible for the induction of the Caspase-3-dependent pyroptotic pathway. At the same time, the canonical inflammasome path is activated by the organic components of PM, including LPS (Lipopolysaccharide/endotoxin).

**Graphical Abstract:**

**Figure:**
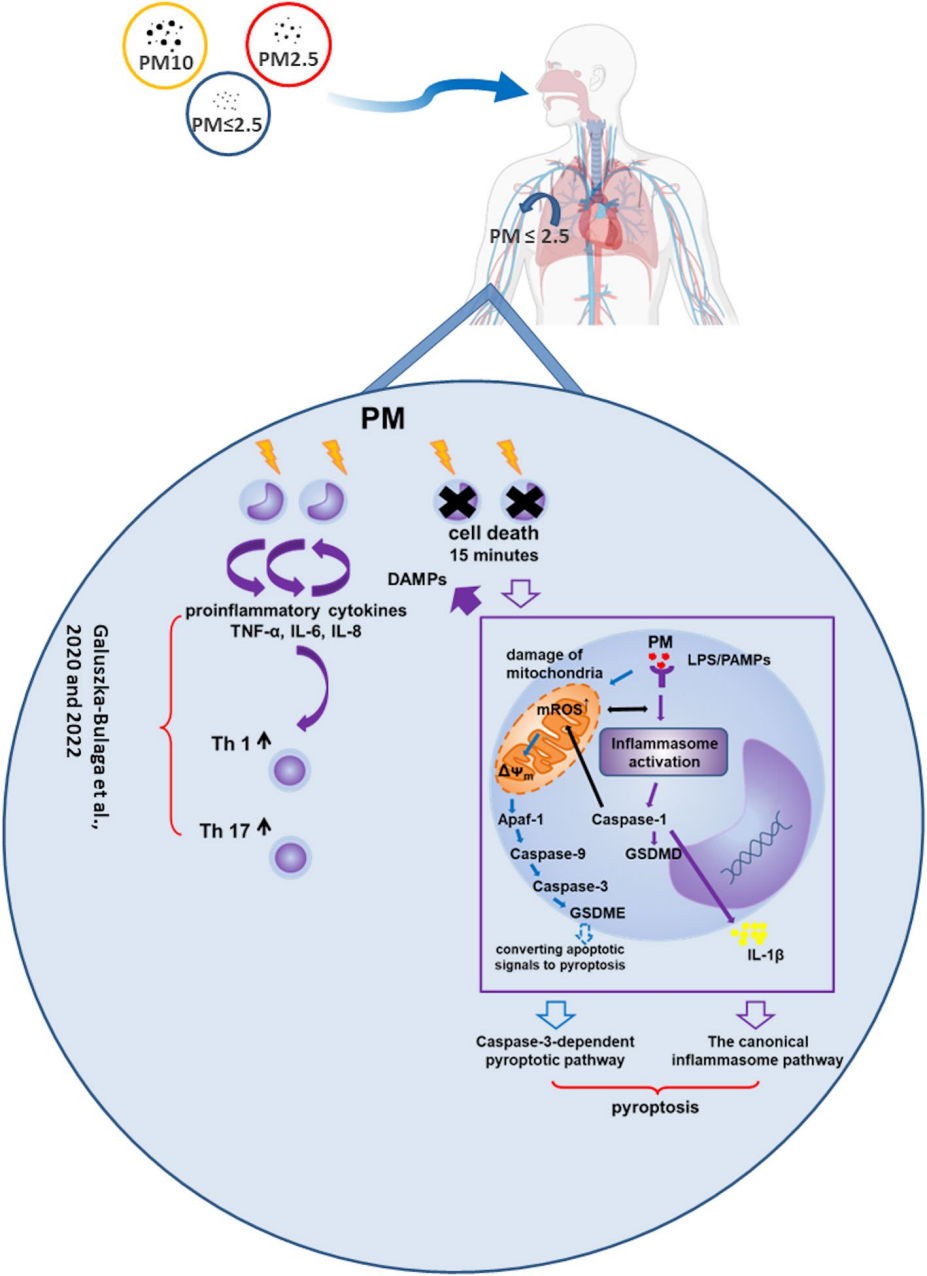
**PM-induced pyroptosis of human monocytes.** Particulate matter (PM) treatment affects monocytes viability already after 15 min of their exposure to NIST or LAP in vitro. The remnant viable monocytes in response to danger-associated molecular patterns (DAMPs) release pro-inflammatory cytokines and activate Th1 and Th17 cells. The mechanism of PM-induced cell death includes the increase of reactive oxygen species (ROS) production followed by collapse of mitochondrial membrane potential (ΔΨ_m_), activation of Apaf-1, Caspase-9 and Caspase-3, leading to activation of Caspase-3-dependent pyroptotic pathway, where Caspase-3 cleaves Gasdermin E (GSDME) to produce a N-terminal fragment responsible for the switch from apoptosis to pyroptosis. At the same time, PM activates the canonical inflammasome pathway, where activated Caspase-1 cleaves the cytosolic Gasdermin D (GSDMD) to produce N-terminal domain allowing IL-1β secretion. As a result, PM-treated monocytes die by pyroptosis activated by two parallel pathways—Caspase-3-dependent pathway related to the inorganic fraction of PM and the canonical inflammasome pathway dependent on the organic components of PM.

**Supplementary Information:**

The online version contains supplementary material available at 10.1186/s12950-023-00353-y.

## Introduction

Despite the growing public awareness and efforts of policy makers to reduce the concentration of particulate matter (PM) in the most polluted cities, air pollution remains one of the most critical factors affecting human health, causing deterioration of quality of life, and increasing social burden. Exposure to PM in combination with genetic predispositions and epigenetic factors may play a role in the initiation and development of civilization diseases, including allergies, autoinflammatory, and autoimmune disorders [1–6]. Moreover, inhaled PM is associated with the risk of pulmonary and cardiovascular disease morbidity and mortality [7–12].

Fine, ultrafine, and nanoparticles contain transition metals (TM), which can modulate activity of the immune system [13–15]. Moreover, air pollution containing TM has different toxic effects [16], depending on the composition, shape, and size of PM, their concentration [17, 18], and source [19, 20]. TM induces local formation of reactive oxygen species (ROS) [21, 22], which play a prominent role in development of many pathologies [23]. ROS formation is associated with changes in mitochondrial membrane potential [24], an indicator of the functional status of mitochondria and the cell, as a whole [25]. Loss of the mitochondrial membrane potential leads to the release of pro-apoptotic components, such as caspase activators (cytochrome c or apoptosis-inducing factor), which are responsible for the activation of Caspase-9 and Caspase-3, causing apoptotic cell death [26]. However, according to recent data, Caspase-3 activation may induce a cleavage of Gasdermin E (GSDME), causing a switch from apoptosis to pyroptosis [27].

Pyroptosis is an inflammatory cell death which may be induced via non-canonical or canonical inflammasome signaling pathways [28]. The non-canonical inflammasome pathway in humans is initiated by activation of Caspases-4/5 [28], whereas the canonical inflammasome pathway is associated with Caspase-1 activation [28]. Inflammasomes are cytosolic complexes, containing Caspase-1, the adaptor protein ASC (apoptosis-associated speck-like protein containing a CARD), and NLRP (nucleotide-binding oligomerization domain-like receptor pyrin domain-containing) [29, 30]. These multi-protein oligomer platforms play an important role in initiating and sustaining inflammation [31]. The well-known types of inflammasomes include AIM2 (absent in melanoma 2), and NLRP3, NLRP6, NLRC4 and NLRP1b, which are activated through different stimuli [31–42]. The main function of inflammasomes is the activation of Caspase-1, which cleaves cytosolic Gasdermin D (GSDMD) to a N-terminal domain (GSDMD-N) and a C-terminal domain (GSDMD-C). GSDMD-N may initiate pyroptosis through oligomerization and formation of transmembrane pores, allowing IL-1β and IL-18 secretion [43]. As a result of the exposure to pro-inflammatory components, a metabolic reprogramming occurs in the immune cells [44]. Inflammatory stimuli switch cellular glucose metabolism from oxidative phosphorylation (OXPHOS) to glycolysis [45, 46], which is also involved in inflammasome activation [47].

Inhaled PM may be deposited in the lower respiratory tract, where it may have a toxic effect on the local cells [16, 48]. A smaller PM can directly translocate from the lungs to the bloodstream [49], where may affect the function of blood erythrocytes, platelets, and leukocytes [50–53]. Monocytes are leukocytes from the first line of defense, able to phagocytose bacteria and other particles, possessing antigen-presenting capacity for T cells, and producing a wide range of humoral factors [54]. They play an important role in the regulation of chronic inflammation associated with infections and other disorders [55]. The role of monocytes in the immune response to conventional antigens is well documented, however, knowledge of the effects of PM containing TM and organic components on the lifespan of human monocytes is scarce.

Considering our previous results suggesting the role of air pollution in the stimulation of pro-inflammatory response of Th1 and Th17 cells, this study provides evidence that short-term exposure of monocytes to PM induces their pyroptosis through two independent pathways and enhances production of pro-inflammatory cytokines by remnant viable cells, leading to initiation of strong inflammatory reaction.

## Material and methods

### PM preparation

SRM 1648a (standard reference material) was supplied by the National Institute of Standards and Technology (NIST, Gaithersburg, MD, USA). SRM 1648a is a conglomeration of fine and ultrafine particles (with high levels of transition metals (TM): iron (Fe) and zinc (Zn)) with a mean particle diameter 5.85 µm. According to the certificate of analysis it contains also organic compounds, including polycyclic aromatic hydrocarbons, polychlorinated biphenyls, and chlorinated pesticides [56]. Our own additional analysis also documented in the NIST particles high content of LPS (Lipopolysaccharide/endotoxin) [57]. SRM 1648a was also treated for 120 min with low-temperature (cold) plasma using a Plasma Zepto system (Diener Electronic GmbH, Germany) at the highest power (100 W) to remove organic compounds from the reference material (hereinafter referred to as LAP). The carbon, hydrogen, nitrogen, and sulfur contents were evaluated by elemental analysis (Elementar, Vario Micro Cube, Germany). The carbon and organic carbon contents in the NIST and LAP samples were determined using a total organic carbon analyzer (Shimadzu, TOC-V series with a Total Nitrogen accessory, Japan). Additionally, a general physicochemical analysis of PM was performed by our partners from the Department of Inorganic Chemistry, Jagiellonian University, Cracow, Poland. NIST contains ca. 13% carbon, including 10.5% organic carbon, and less than 2% organic carbon, after the removal of organic compounds from the reference material (LAP) [58]. Similarly, the LPS content was significantly lower than in the NIST particles [57]. For in vitro culture, PM preparations were weighed on a high-precision microbalance and freshly suspended in RPMI (Roswell Park Memorial Institute) 1640 medium (Corning, Manassas, VA, USA) under sterile conditions. The final concentrations of PM (1, 10, and 100 µg/mL) in monocyte cultures were established experimentally, based on our [57] and other in vitro studies [59–61].

### Monocytes isolation and cell culture

CPDA-treated blood samples from healthy donors were purchased from the Regional Center of Blood Donation and Blood Therapy in Krakow, Poland. Peripheral blood mononuclear cells (PBMCs) were isolated using standard Pancoll human (Panbiotech, Aidenbach, Germany) density gradient centrifugation. Monocytes were separated from PBMCs by counter-current centrifugal elutriation (JE-6B elutriation system equipped with a 5-mL Sanderson separation chamber; Beckmann-Coulter, Palo Alto, CA, USA), as described previously [62]. The cells were washed and resuspended in RPMI 1640 medium (Corning) and kept in an ice bath until use. The purity of isolated monocytes was confirmed by flow cytometry using an anti-CD14 antibody (BD Biosciences Pharmingen, San Diego, CA, USA) and did not drop below 90%.

Monocytes (5 × 10^5^/mL) were cultured in Ultra Low Attachment tubes (Corning) in RPMI 1640 medium (Corning) supplemented with 2 mM L-glutamine, phenol red, 5–10% heat-inactivated fetal bovine serum (FBS, EURx, Gdańsk, Poland) and 25 µg/mL gentamycin (Sigma, St. Louis, MO, USA) (complete medium), with or without NIST and LAP at three concentrations (1, 10, and 100 µg/mL). Cells were kept at 37 °C in a humidified atmosphere with 5% CO_2_ and collected after 15 min, 2 and 4 h of culture. The time points of the analysis were experimentally established during the cell culture. For some experiments, a population of lymphocytes containing approximately 80% CD3-positive cells was isolated by counter-current centrifugal elutriation from PBMCs.

Additionally, in some experiments, monocytes were simulated with 10 µM CCCP (15 min, carbonyl cyanide m-chlorophenylhydrazone, Sigma), an uncoupler of oxidative phosphorylation; 200 µM TBHP (tert-butyl hydroperoxide, Invitrogen, Carlsbad, CA, USA); 100 ng/mL of LPS (lipopolysaccharide) from *Salmonella abortus equi* (Sigma); or 400 U/mL of human recombinant IFN-γ (Sigma) with 100 ng/mL of LPS from *Salmonella abortus equi* (Sigma) (positive controls). As a negative control, a 1 h cell pre-incubation with the inhibitor of mitochondrial ROS production (1.5 mM; Mito-TEMPO ((2-(2,2,6,6-Tetramethylpiperidin-1-oxyl-4-ylamino)-2-oxoethyl) triphenylphosphonium chloride), Sigma) or Caspase-1 inhibitor (50 µM; Ac-yvad-cmk; InvivoGen, San Diego, CA, USA), was done prior to PM exposure. In the experiments assessing the role of phagocytosis in PM internalisation, monocytes were preincubated (1 h) with cytochalasin D (0.1 μM; Sigma) or were incubated with PM on ice. Additionally, polyclonal mouse anti-human TLR4 antibody and relevant isotype control (both 5 μg/mL; InvivoGen) were used to block LPS binding. To block NLRP3 activation, a selective inhibitor – N-((1,2,3,5,6,7-heksahydro-s-indacen-4-ylcarbamoyl)-4-(2-hydroxy-2-propanyl)-2-furansulfonamide) (MCC950; 10 μM; Sigma) was used prior to PM stimulation.

### Antigen pulse and T-cell proliferation

T cell proliferation in response to PPD (purified protein derivative of tuberculin) antigen (Statens Serum Institute, Copenhagen, Denmark) stimulation was analyzed, as described previously [63]. Briefly, monocytes at the density of 4 × 10^5^/mL suspended in DMEM (Dulbecco's Modification of Eagle's Medium) medium (Corning), containing phenol red and supplemented with 2 mM L-glutamine, 10% human AB serum (Sigma), and 25 µg/mL gentamycin (Sigma) – complete DMEM medium were exposed (15 min) in Ultra Low Attachment tubes (Corning) to NIST or LAP used in three concentrations: 1 µg/mL, 10 µg/mL, and 100 µg/mL. Thereafter, control samples (monocytes not exposed to PM) and monocytes exposed to PM were divided into two parts: one part was pulsed with a “recall” PPD at a concentration of 100 µg/mL, while the second part of monocytes was incubated with no antigen (additional control). For these experiments only blood donors responding for PPD were selected. After a 2 h antigen pulse (37 °C, humidified atmosphere with 5% CO_2_), monocytes were washed with complete DMEM medium. Four groups of monocytes (control and control PPD-pulsed, PM, and PM PPD-pulsed) at a density of 1 × 10^4^/mL were cultured for 7 days (37 °C, 5% CO_2_ in a humidified atmosphere) in complete DMEM medium (0.2 mL) in flat-bottom 96-well plates (Corning) with 1 × 10^5^/mL of autologous T lymphocytes. ^3^H-thymidine (Hartmann Analytic GmbH, Braunschweig, Germany) (1 µCi per well) was added for overnight incubation. Subsequently, cultures were harvested, and cell radioactivity was measured using a Beckman LS1801 Scintillation Beta Counter (Beckman-Coulter, Brea, CA, USA). Results were expressed as counts per minute (cpm), and proliferation index was calculated as a ratio of cpm from T cell cultures with antigen-pulsed monocytes to cpm from T cell cultures with monocytes without antigen pulse.

### Cell morphology

To estimate the morphological changes in monocytes after 15 min, 2 and 4 h exposure to PM, 2 × 10^5^/mL cells per glass slide were spun in a Cytospin 2 centrifuge (Shandon, England) for 5 min, fixed in methanol, and stained with May-Grünwald-Giemsa dye (Sigma). Cells that were not exposed to PM were used as controls. Monocyte morphology was assessed by light microscopy (Olympus BX53, Olympus Corporation, Tokyo, Japan), using 100x/1.40 magnification (Oil objective, Olympus). Images were processed using CellSens Dimension v.1.16 software (Olympus).

### Monocyte viability 

Monocyte viability was assessed by flow cytometry using Annexin-V Apoptosis Detection Kit I (BD Pharmingen), according to the manufacturer’s instructions. Briefly, monocytes after 15 min, 2 and 4 h of culture with or without PM were harvested, washed in PBS (Corning), resuspended in binding buffer, and stained with Annexin V-FITC (15 min at room temperature in the dark). The stained cells were examined using flow cytometry (FACSCalibur, BD Biosciences). Typically, 10,000 monocytes were acquired for analysis. Additionally, monocyte viability and Annexin-V binding was determined after Mito-TEMPO (Sigma) or Ac-yvad-cmk (InvivoGen) pre-treatment prior to 15 min of PM exposure and after 15 min of incubation with CCCP (positive control; Sigma). Moreover, in some experiments, monocytes after 2 h exposure to PM were re-challenged with the same dose of PM for an additional 15 min or 2 h. Thereafter, cell viability was determined (as described above). The results are expressed as a percentage of Annexin V-FITC-positive cells.

### ROS production

Monocytes (5 × 10^4^/mL) in 50 µl of complete medium with or without PM and 100 µl of 2 mM luminol (5 amino-2,3 dihydro-1,4 phthalazinedione) (Sigma) in Krebs–Ringer buffer (1.12 mM Mg2 + ; 0.54 mM Ca2 +) and 11 mM glucose (Sigma) were suspended in 96-well White Opaque Tissue Culture Plates (Falcon, Corning, NY, USA). The plates were placed in the measuring chamber of a Victor^2^ plate reader (EG&G WALLAC, Turku, Finland) at 37 °C, and ROS formation was analyzed using luminol-dependent chemiluminescence measurements. The results were expressed as cumulative counts of responses recorded for 100 min from 60 cycles. Additionally, detection of ROS in monocytes was performed by flow cytometry using a FACSCanto X flow cytometer (BD Biosciences) after cell staining with 1 µM CellROX Green reagent (Invitrogen). The results are expressed as a frequency of CellROX Green positive cells.

###  Mitochondrial membrane potential (ΨMMP)

To determine changes in the mitochondrial membrane potential (*ΔΨMMP*) of monocytes after exposure to PM, cells were stained with MitoScreen JC-1 dye (5,5′,6,6′-tetrachloro-1,1′,3,3′-tetraethylbenzimidazolyl-carbocyanine iodide; BD Pharmingen) for 15 min at 37 °C, according to the manufacturer’s instructions. Thereafter, cells were washed twice in warm washing buffer and *ΨMMP* was analyzed by flow cytometry (FACSCalibur, BD Biosciences) after 15 min of monocyte exposure to PM. Data from 10,000 monocyte events were recorded and analyzed using CellQuest v.3.1 software (BD Biosciences). The relative changes in *ΨMMP* were determined as the ratio of aggregates (high fluorescence intensity) to monomers (low fluorescence intensity).

### Cellular respiration

For the assessment of mitochondrial respiration, monocytes were seeded at 150,000 cells/well in XF^e^ 8 well cell culture microplates (Agilent Technologies, Palo Alto, CA, USA), covered with a combination of laminin, fibronectin, and poly-l-lysine (all from Sigma) in 80 µl of complete medium. Cells were kept in microplates at 37 °C and 5% CO_2_ in a humidified atmosphere overnight to allow them adherence to the plastic. Thereafter, 60 µl of supernatant was discarded and 180 μl of the Seahorse media (XF-based minimal DMEM; Agilent Technologies) supplemented with 5.5 mM glucose (Sigma), 1 mM Sodium Pyruvate (Gibco, Gaithersburg, MD, USA), and 2 mM L-glutamine (Gibco) were carefully added to avoid disturbing the cell layer. The XF^e^ 8 well cell culture microplates were incubated at 37 °C in a non-CO_2_ incubator for 1 h. The assay sensor cartridge was hydrated overnight with 200 μl of water for injection at 37 °C (in a non-CO_2_ incubator). Thereafter, water was discarded and replaced with 200 µl of XF Calibrant Media (Agilent Technologies) for at least 3 h. Before the measurement of OCR (oxygen consumption rate), PM was added directly to XF^e^ 8 well cell culture microplate with monocytes, Oligomycin (1 µM; Agilent Technologies) was added to Port A of the assay cartridge, FCCP (2 µM; carbonyl cyanide-4-(trifluoromethoxy)phenylhydrazone, Agilent Technologies) was added to Port B and Rotenone/antimycin A (0.5 µM; Agilent Technologies) were added to Port C (final concentrations). Optimization of cell density and Oligomycin and FCCP concentrations were experimentally established. Key parameters of mitochondrial function, OCR, basal, maximal, and non-mitochondrial respiration, ATP production, proton leak, and spare capacity were measured using a Seahorse XFp Extracellular Flux Analyzer (Agilent Technologies) using Seahorse XFp Cell Mito Stress Test (Agilent Technologies), according to the manufacturer’s instructions, and analyzed by Wave Desktop and Controller 2.6 Software (Agilent Technologies).

### Western blotting

For Western blot analysis, monocytes were harvested after a 15 min exposure to PM, washed in PBS (Corning), and lysed in Mammalian Protein Extraction Reagent (Sigma) containing protease inhibitor (Sigma). Cell-free supernatants were collected for protein analysis. Equal amounts of proteins were loaded onto 10% polyacrylamide gels, resolved by SDS-PAGE, and transferred to PVDF membranes. PageRuler™ Plus Prestained Protein Ladder, 10 to 250 kDa, (catalog number: 26620; Thermo Fisher Scientific Inc., Waltham, MA, USA), was used as size standards in SDS-PAGE and Western blotting. Next, membranes were incubated for 1 h with 5% BSA (bovine serum albumin; Sigma) in tris-buffered saline with 0.05% Tween 20 (Sigma) to block non-specific binding and washed before the overnight incubation with primary rabbit monoclonal antibodies: anti-Apaf-1 (clone D7G4; 1:1000), anti-NLRP3 (clone D4D8T; 1:1000), anti-Gasdermin E (clone E2X7E; 1:1000) and anti-Gasdermin D (clone E8G3F; 1:1000, all from Cell Signaling Technology, Danvers, MA, USA) at 4 °C, followed by 1 h incubation at RT with secondary anti-rabbit IgG HRP-conjugated antibody (1:5000; Cell Signaling Technology). Blotting with rabbit anti-GAPDH monoclonal antibody (D16H11) (1:5000; Cell Signaling Technology) was performed to confirm the equivalent protein loading. Protein expression was detected using SuperSignal™ West Pico PLUS Chemiluminescent Substrate (Thermo Fisher Scientific Inc.) and a ChemiDoc Imaging System (Bio-Rad, Warsaw, Poland).

###  Caspase-1, Caspase-3, and Caspase-9 activity

Caspase-1 activity was detected after 15 min, 2 and 4 h of monocyte incubation with or without PM using a Caspase 1 (active) Staining Kit (Abcam, Cambridge, MA, USA), according to the manufacturer’s instructions. Cells were harvested and stained with FAM-YVAD-FMK in the dark at 37 °C in a humidified atmosphere with 5% CO_2_ for 1 h. The stained cells were immediately examined for green fluorescence of FAM-YVAD-FMK by flow cytometry (FACSCanto X, BD Biosciences).

Caspase-3 activity was determined at the same time points of monocyte exposure to PM. Cells were harvested and washed in PBS (Corning) with 2% FBS (EURx), fixed and permeabilized with Cytofix/Cytoperm solution (BD Biosciences), according to manufacturer’s instructions. Then, the cells were washed twice in Perm/Wash solution (BD Biosciences) and stained (30 min at 4 °C, dark condition) for active Caspase-3 using PE-conjugated monoclonal antibody (BD Pharmingen). After staining cells were washed twice in PBS with 2% FBS and suspended in PBS for flow cytometry analysis (FACSCanto X, BD Biosciences).

In some experiments after 2 h of monocyte exposure to NIST or LAP, cells were additionally re-challenged with the same dose of PM for next 15 min or 2 h and stained for Caspase-1 and Caspase-3 activity (as above).

Caspase-9 activity was assessed after 15 min, 2 and 4 h of monocyte incubation with or without PM using an active Caspase-9 FITC Staining Kit (Abcam). For this purpose, cells were harvested and stained with FITC-LEHD-FMK in the dark at 37 °C in a humidified atmosphere with 5% CO_2_ for 45 min. The stained cells were immediately examined using a FACSCanto X flow cytometer (BD Biosciences) and analyzed for green fluorescence of FITC-LEHD-FMK by CellQuest v.3.1 software (BD Biosciences).

### Immunostaining for intracellular IL-1β

Monocytes (3 × 10^5^/mL) were cultured in Ultra Low Attachment tubes (Corning) in complete medium with or without PM, at 37 °C in a humidified 5% CO_2_ atmosphere. For intracellular detection of IL-1β, 2 µM Golgi Stop (containing monensin; BD Biosciences) was added at the beginning of culture. After 15 min and 4 h of the culture, cells were harvested, washed in PBS with 2% FBS, fixed and permeabilized with Cytofix/Cytoperm solution. Next, cells were washed twice in Perm/Wash solution and stained (30 min at 4 °C, dark) for intracellular cytokine using a PE-labelled mouse anti-human IL-1β monoclonal antibody (BD Pharmingen). The IL-1β producing monocytes were analyzed using a FACSCanto X flow cytometer with BD FACSDiva v. 8.0.1 software. In some experiments, monocytes after the first exposure to PM for indicated time were re-challenged with the same doses of PM for additional 2 h. Thereafter, the level of IL-1β positive cells was determined (as above).

### Cytokine detection by CBA

Monocytes (3 × 10^5^/mL) were cultured at 37 °C in a humidified, 5% CO_2_ atmosphere in Ultra Low Attachment tubes (Corning) in complete medium with or without PM in three concentrations (1 µg/mL, 10 µg/mL and 100 µg/mL). Supernatants from monocyte cultures were collected after 4 h and the concentration of cytokines (IL-1β, IL-6, IL-8 and TNF-α) was determined by Cytokine Bead Array (CBA, Human Inflammatory Cytokine Kit; BD Biosciences), according to manufacturer’s instructions. Typically, data from 3,600 beads were acquired by FACSCanto X flow cytometer and analyzed using FCAP Array v. 3.0 software (BD Biosciences).

### Statistics

The normal distribution of the data was verified using the Shapiro–Wilk test. Statistical analysis was performed using the Kruskal–Wallis test for data without normal distribution and ANOVA for data with normal distribution using PRISM GraphPad 6.01 software (GraphPad Software Inc., San Diego, CA, USA). Data are presented as median with interquartile range. Statistically significant differences were considered at the following *p*-values: *p* < 0.05, *p* < 0.01, *p* < 0.001, and *p* < 0.0001.

## Results

### Monocyte treatment with PM affects T-cell response to recall antigen

**Fig. 1:**
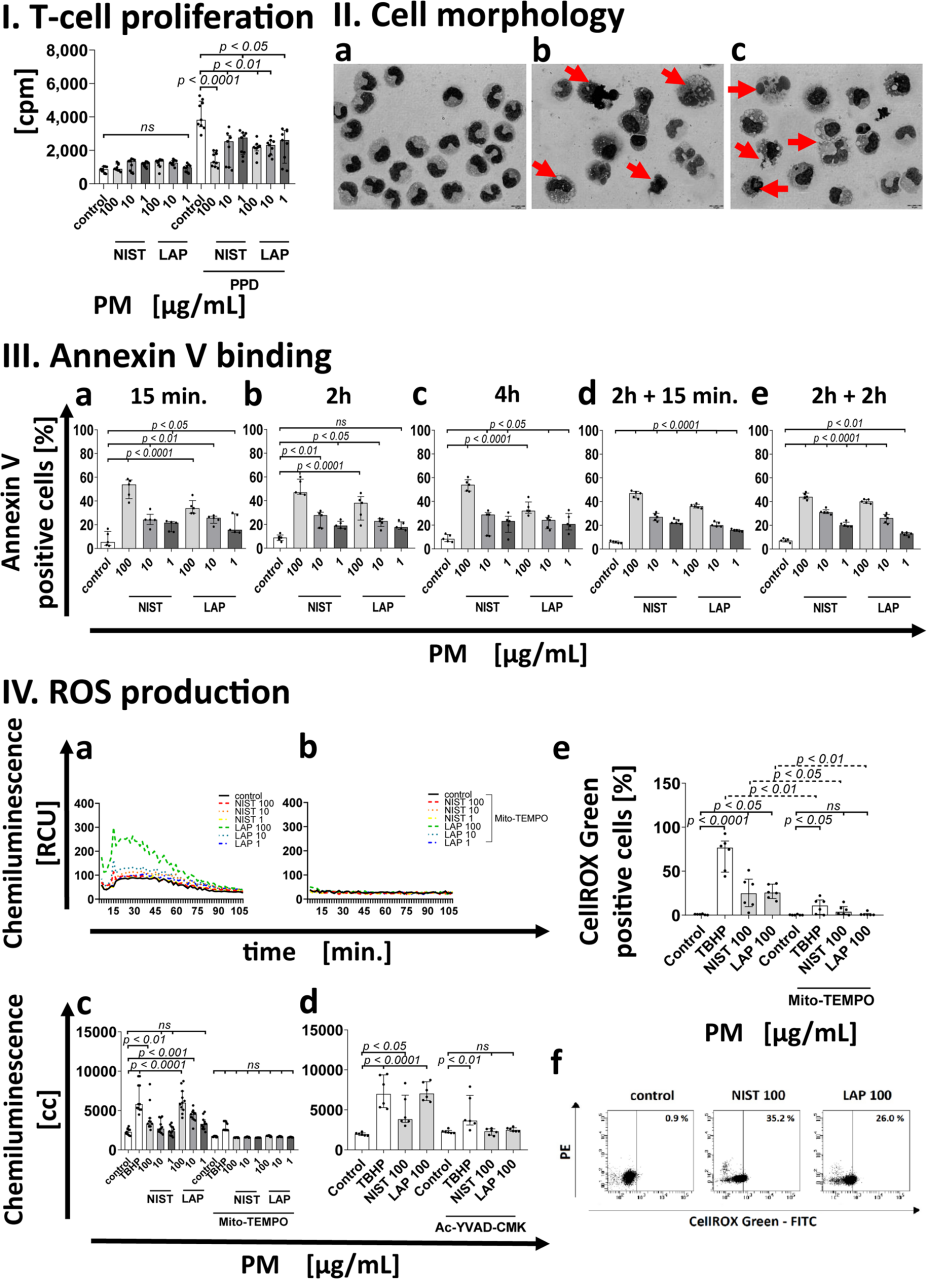

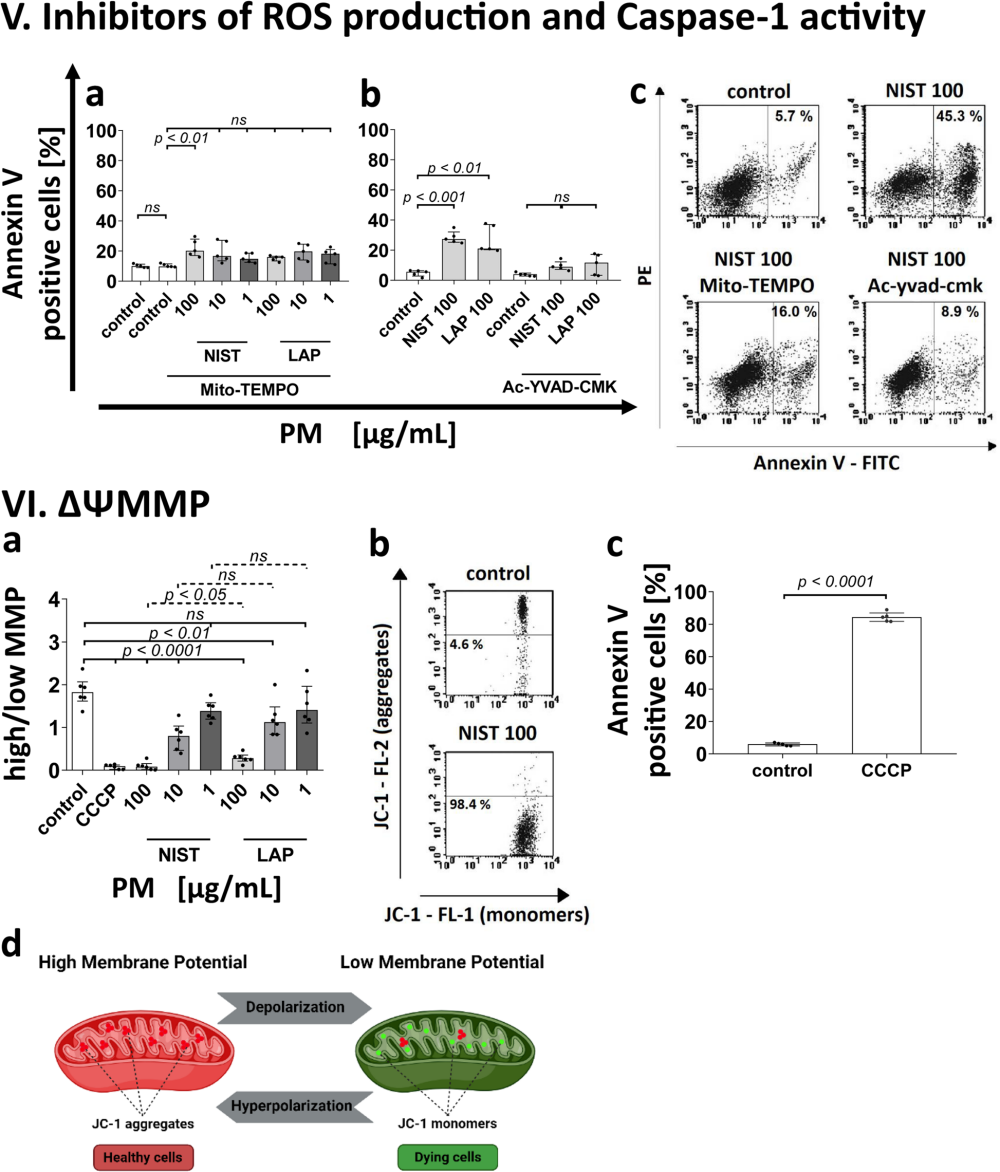
Effect of the PM exposure of monocytes on: (I) Antigen-driven T cell proliferation. Antigen -driven T cell proliferation in the presence of monocytes exposed to NIST or LAP as APC was evaluated by [^3^H]-thymidine incorporation assay after 7 days stimulation with recall antigen PPD (purified protein derivative of tuberculin). Data are presented as counts per minute (cpm; median ± interquartile range from 9 independent experiments). (II) Cell morphology. Images show monocytes without exposition to PM (**a**) vs. exposed to NIST 100 µg/mL (**b**) or LAP 100 µg/mL (**c**). Arrows indicate alterations in cell morphology after 15 min exposure to PM. Data are presented from one representative experiment (May-Grünwald-Giemsa staining; magnification × 1000, scale 10 µm). (III) Annexin V binding. The viability of monocytes was evaluated by Annexin V binding assay and flow cytometry analysis. Data are presented as a percentage of Annexin V positive cells (median ± interquartile range from 5 independent experiments). Cells were cultured with a single dose of NIST, or LAP used in three different concentrations (1, 10 or 100 µg/mL) for 15 min (**a**), 2 h (**b**) and 4 h (**c**). In some experiments, monocytes were cultured with PM for 2 h and then the second dose of NIST or LAP was added to cell cultures for the next 15 min (**d**) or 2 h (**e**). (IV) ROS production. ROS formation was analyzed by luminol-dependent chemiluminescence measurement recorded at 37 °C. Data are presented in relative chemiluminescence units (RCU) as mean of the results of each cycle (from 1 to 60 cycles) (**a**, **b**) and results are expressed as cumulative counts (cc) of the response (**c**, **d**) recorded from 60 cycles during 100 min. of measurement. Data are presented as median ± interquartile range from 11 independent experiments. Additionally, detection of ROS in live monocytes were performed with CellROX Green reagent by flow cytometry after 15 min of the cell exposure to NIST or LAP (**e**). Data are expressed as the percentage of CellROX Green positive cells (median ± interquartile range from 6 independent experiments). Dot plots (CellROX Green—FITC vs. PE) show CellROX Green positive cells in unstimulated control and cells stimulated with NIST or LAP in the highest concentration of 100 µg/mL, for 15 min (**f**). As a positive control, 200 µM TBHP was used. As a negative control, monocytes were pre-treated with 1.5 mM Mito-TEMPO (**c**) or 50 µM Ac-yvad-cmk (**d**) for 1 h prior to the PM exposure. (V) Inhibitors of ROS production and Caspase-1 activity. Additionally, the viability of cells was evaluated by Annexin V binding assay and flow cytometry analysis after monocyte pre-incubation (1 h) with 1.5 mM Mito-TEMPO or 50 µM Ac-yvad-cmk prior to PM exposure (**a**, **b**). Dot plots (Annexin V—FITC vs. PE) show Annexin V positive cells in unstimulated control, cells stimulated with NIST (100 µg/mL) for 15 min and cells treated with 1.5 mM Mito-TEMPO or 50 µM Ac-yvad-cmk for 1 h prior to the NIST (100 µg/mL) exposure (**c**). (VI) Mitochondrial membrane potential (*ΨMMP*). Alteration of mitochondrial membrane potential (*ΔΨMMP*) was appointed by flow cytometry using MitoScreen JC-1 dye after 15 min the exposure of cells to NIST or LAP. *ΔΨMMP* of monocyte was expressed as a ratio of the percentages of cells with high fluorescence intensity (aggregates with high *ΨMMP*) to cells with low fluorescence intensity (monomers with low *ΨMMP*) (**a**). As a positive control, 10 µM CCCP was used. Dot plots (JC-1—FL-1 vs. JC-1 – FL-2) show JC-1 positive cells for aggregates with high *ΨMMP*; high fluorescence intensity and monomers with low *ΨMMP*; low fluorescence intensity in unstimulated control and cells stimulated with NIST (100 µg/mL) for 15 min (**b**). Data are presented as median ± interquartile range from 6 independent experiments. Additionally, the viability of cells was evaluated by Annexin V binding assay and flow cytometry analysis after cell incubation with 10 µM CCCP for 15 min (**c**). Data are presented as a percentage of Annexin V positive cells (median ± interquartile range from 5 independent experiments). Statistically significant differences were estimated at *p* < 0.05, *p* < 0.01, *p* < 0.001, *p* < 0.0001, ns – not significant. (**d**) Changes in mitochondrial membrane potential of cells

The effect of monocyte treatment with PM on the induction of the immune response was assessed by analysis of autologous T-cell proliferation induced by recall antigen PPD. To this end, monocytes after a 15 min exposure to PM used in three different concentrations (1 µg/mL, 10 µg/mL, and 100 µg/mL) were pulsed with PPD for 2 h, washed free of antigen and added to autologous T lymphocytes, and cultured for the next 7 days. Afterwards, T cell proliferation was measured using a ^3^H-thymidyne incorporation assay. Results presented in Table 1 show that the exposure of monocytes to PM significantly reduced the proliferation index of T cells. The index was at the similar level both for PPD presented by monocytes exposed to NIST and LAP particles, being the most impaired when monocytes were exposed to NIST, or LAP used in the highest concentration. As NIST differs from LAP in the content of organic components, which are highly reduced in LAP, this observation suggests that the effect of PM on monocytes is dependent on both the organic and inorganic fraction content in PM (Fig. 1 I).

**Table 1:** Antigen driven T-cell proliferation after monocyte exposure to NIST or LAP particles

	**Control**	**PM [µg/mL]**					
		**NIST**			** LAP**		
		**100**	**10**	**1**	**100**	**10**	**1**
**proliferation** **index**^***a***^	4.89	1.50	1.90	2.07	1.74	1.95	2.47

^*a*^proliferation index – expressed as a ratio of cpm from T cell cultures with monocytes after PPD-pulse to cpm from T cell cultures with monocytes without PPD-pulse

### PM exposure alters monocyte morphology and induces Annexin-V binding

Decrease in the antigen-driven proliferation of T cells due to monocyte exposure to PM prompted us to the assessment of the effects of PM on monocyte morphology and viability. Monocyte morphology was analyzed by phase-contrast microscopy and morphological alterations were detected already after 15 min treatment with NIST and LAP (Fig. 1, panel II). This effect was observed both for NIST (Fig. 1 IIb) and LAP (Fig. 1 IIc), being most evident for the highest concentrations (100 µg/mL) of PM. After exposure to PM, many monocytes presented swelling and disintegration of cytoplasm. The cell nuclei with no cytoplasm and lack of the cell membrane integrity were also observed. In the case of monocytes treated with LAP, analysis of their morphology additionally revealed the formation of numerous vacuoles in the cytoplasm, not observed in the group of cells treated with NIST (Fig. 1 IIc).

Detected alterations in monocyte morphology after NIST or LAP treatment was accompanied by the increase of Annexin-V binding (Fig. 1, panel III), indicating a cell death. This effect was observed for both PM preparations, being more significant for their highest concentration. Moreover, the level of Annexin-V positive monocytes persisted at the similar level for a 4 h PM treatment, suggesting that not all monocytes reacted to PM in the same manner (Fig. 1, IIIa, IIIb and IIIc). In this context, it was relevant to answer whether monocytes which survived the first contact with PM could become resistant to additional exposure to NIST or LAP. To this end, monocytes were re-challenged with the same doses of PM for next 15 min (Fig. 1 IIId) or 2 h (Fig. 1 IIIe). The obtained data showed that percentage of Annexin-V positive cells did not differ significantly when compared to the level observed after 2 (Fig. 1 IIIb) and 4 h (Fig. 1 IIIc) treatment with a single dose of NIST or LAP.

### PM induces ROS production in monocytes

Annexin-V binding indicated monocyte death after the PM treatment. In this context, the potential involvement of ROS was investigated. The formation of ROS was analyzed by kinetic luminol-dependent chemiluminescence measurement for 100 min after the addition of PM to the cell culture (Fig. 1, panel IV). The obtained results showed that ROS were produced in high amounts during the first 15 min (Fig. 1 IVa) after PM were added to monocyte cultures and this production slowly decreased in time (Fig. 1 IVa). Moreover, ROS production was dependent on the dose of PM, being the strongest for LAP used in the highest concentration (Fig. 1 IVc and IVd).

Additionally, the formation of ROS was confirmed by flow cytometry using CellROX Green reagent, which detects ROS in mitochondria. The results documented an increase in the frequency of cells positive for CellROX Green, proving that air pollution induces ROS mainly in mitochondria (Fig. 1 IVe and IVf). ROS can induce cell death both by apoptosis through the intrinsic mitochondrial pathway or by pyroptosis through the activation of inflammasomes, including NLRP3, and Caspase-1. Both forms of cell death are characterized by Annexin-V binding. In the next step, to determine in cell death the role of ROS produced by monocytes exposed to NIST or LAP, a Mito-TEMPO—an inhibitor of mitochondrial ROS production (Fig. 1 IVb and IVc), and Ac-yvad-cmk – an inhibitor of Caspase-1 activity (Fig. 1 IVd), were used prior to cell exposure to PM. The obtained results showed that pre-incubation of cells with these inhibitors efficiently improved viability of monocytes confirmed by Annexin-V binding, even when NIST particles were used in the highest concentration (Fig. 1 Va, Vb and Vc).

### PM treatment decreases the mitochondrial membrane potential (ΨMMP)

To answer whether ROS produced by monocytes exposed to PM affect the mitochondrial membrane potential (*ΨMMP*), the cells were stained with JC-1 dye and analyzed by flow cytometry. Data revealed that PM treatment changed *ΨMMP* already 15 min after the exposure to NIST or LAP, in a dose dependent manner (Fig. 1, panel VI). Similar pattern was observed for both types of particles, being predominant for NIST (Fig. 1 VIa).

### Air pollution affects monocyte mitochondrial respiration

**Fig. 2:**
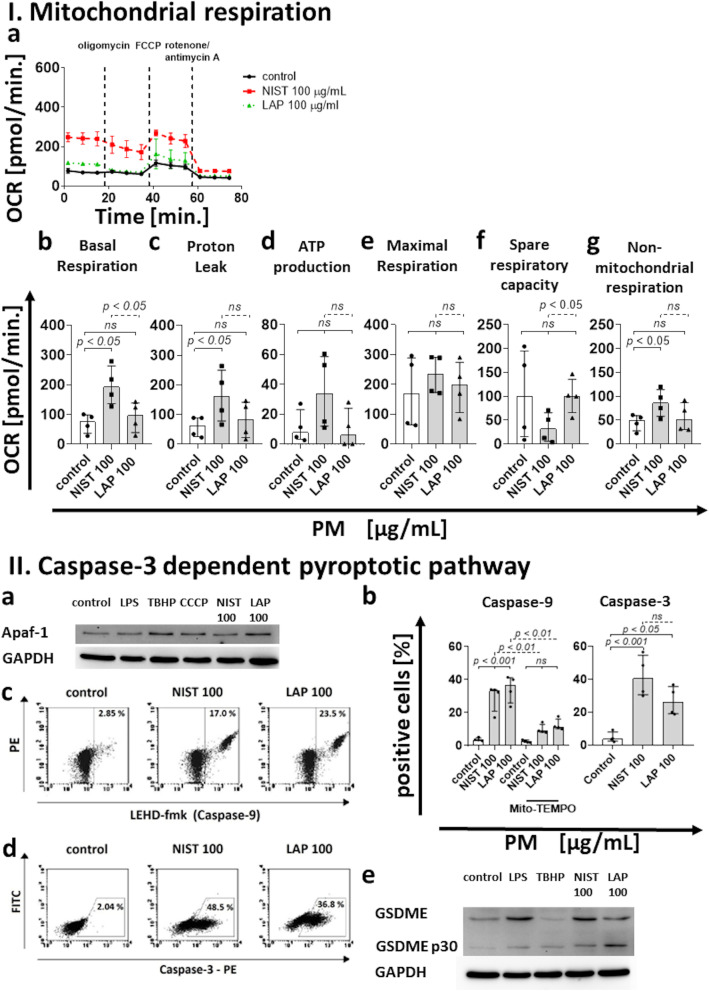

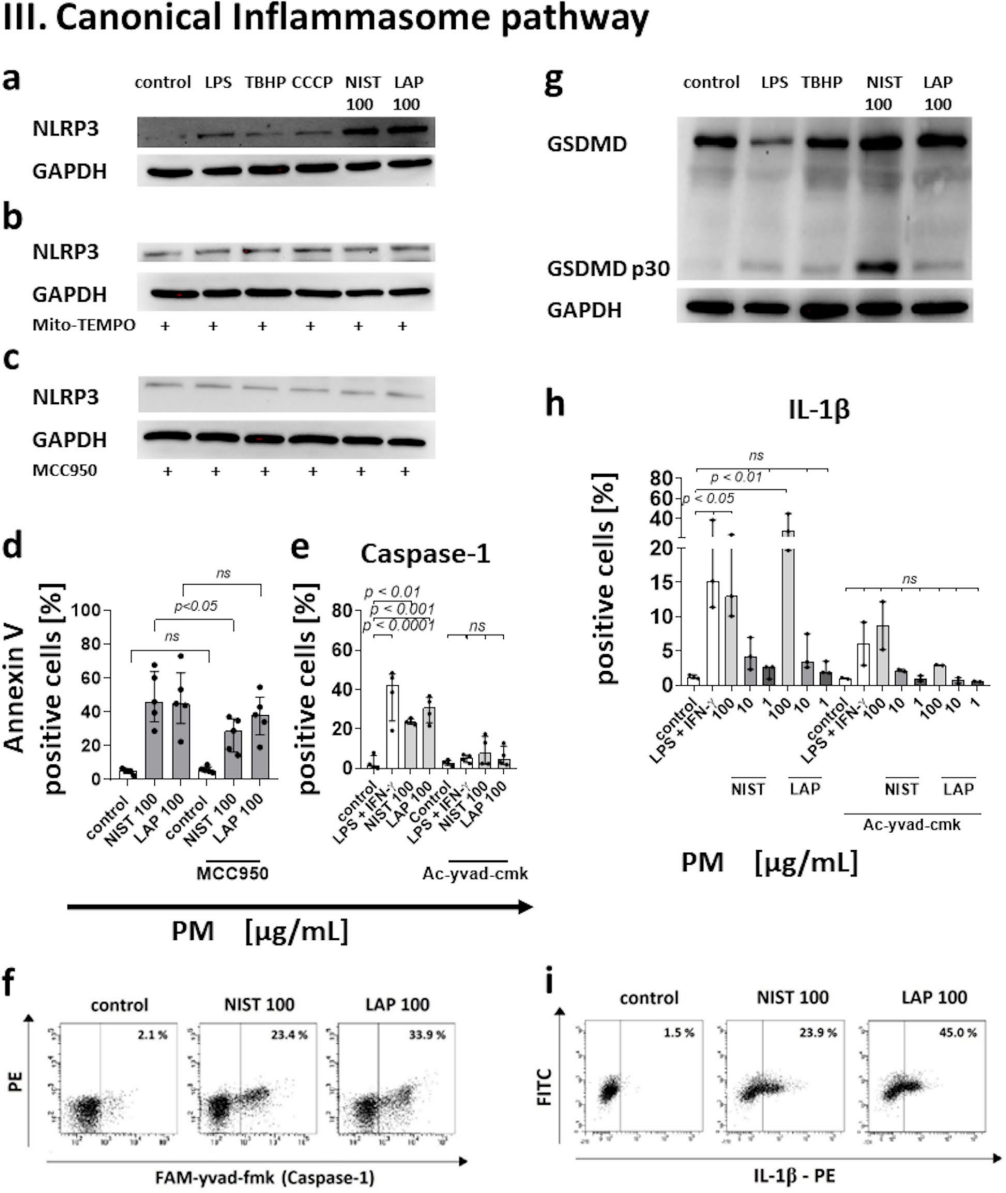
Effect of the PM exposure of monocytes on: (I) Mitochondrial respiration. (**a**) The Cell Mito Stress Test profile presenting OCR values for monocytes in control condition and after NIST or LAP treatment (data are presented from one representative experiment). Additionally, OCR values for: (**b**) basal respiration, (**c**) proton leak, (**d**) ATP production, (**e**) maximal respiration, (**f**) spare respiratory capacity and (**g**) non-mitochondrial respiration in control cells and monocytes treated with NIST or LAP (100 µg/mL) are presented separately, as median ± interquartile range from 4 independent experiments. All measurements were performed in triplicates. (II) Caspase-3–dependent pyroptotic pathway. (**a**) Activation of Apaf-1 and (**e**) GSDME after 15 min of monocyte exposure to NIST or LAP (100 µg/mL) was evaluated by Western blot analysis. GAPDH was used to confirm the equal protein loading. (**b**) In the case of Caspase-9 and Caspase-3, their activation was examined by flow cytometry using Caspase-9 (active) Staining Kit and PE-conjugated mouse anti-human active Caspase-3 monoclonal antibodies. Data are presented as median ± interquartile range from 4 independent experiments. (**c**, **d**) Dot plots (LEHD-fmk – FITC vs. PE and Caspase-3 – PE vs. FITC) show cells positive for Caspase-9 and Caspase-3 in unstimulated control and cells stimulated with NIST or LAP (100 µg/mL) for 15 min. As a positive control, cells stimulated with 100 ng/mL of LPS from *Salmonella abortus equi*, 200 µM TBHP or 10 µM CCCP, were used simultaneously. Monocytes pre-incubated for 1 h with 1.5 mM Mito-TEMPO prior to PM exposure were used as a negative control. (III) The canonical inflammasome pathway. (**a**) Activation of NRLP3, including after 1 h pre-incubation with1.5 mM Mito-Tempo (**b**) or 10 µM MCC950 (**c**) and GSDMD (**g**) was evaluated by Western blot analysis after 15 min of monocyte exposure to NIST or LAP (100 µg/mL). GAPDH was used to confirm the equal protein loading. (**d**) Specific inhibition of NLRP3. MCC950 was used to assess the effect of NLRP3 inactivation on monocyte Annexin V binding. (**e**) In the case of Caspase-1, its activation was examined by flow cytometry using Caspase-1 (active) Staining Kit. Data are presented as median ± interquartile range from 4 independent experiments. (**f**) Dot plots (FAM-yvad-fmk vs. PE) show cells positive for Caspase-1 in unstimulated control and cells stimulated with NIST or LAP (100 µg/mL) for 15 min. (**h**) Additionally, the level of IL-1β producing monocytes was evaluated by flow cytometry after cell staining with a PE-conjugated mouse anti-human IL-1β monoclonal antibodies. Human monocytes were cultured with or without NIST or LAP for 15 min. Data are presented as a percentage of IL-1β positive cells (median ± interquartile range from 3 independent experiments). (**i**) Dot plots (IL-1β – PE vs. FITC) show cells positive for IL-1β in unstimulated control and cells stimulated with NIST or LAP (100 µg/mL) for 15 min. As a positive control, cells stimulated with 100 ng/mL of LPS from *Salmonella abortus equi* and 400 U/mL of human recombinant IFN-γ, 200 µM TBHP or 10 µM CCCP, were used simultaneously. Monocytes pre-incubated for 1 h with 1.5 mM Mito-TEMPO or 50 µM Ac-yvad-cmk prior to PM exposure were used, as a negative control. Statistically significant differences were estimated at *p* < 0.05, *p* < 0.01, *p* < 0.001, *p* < 0.0001, ns – not significant

The oxygen consumption rate (OCR) of monocytes treated with NIST or LAP indicated the changes in mitochondrial respiration, with the biggest variations observed for NIST (Fig. 2 Ia). In this context, the measurements at the basal respiration phase showed the increase in OCR values in monocytes exposed to NIST, suggesting that these particles enhance the initial cellular energy requirements (Fig. 2 Ib). Moreover, the results indicated the increase of proton leak, which is considered as a marker of mitochondrial damage (Fig. 2 Ic). The OCR values recorded during the ATP production phase after monocyte exposure to NIST suggested a higher cell demand for energy (Fig. 2 Id). Increased oxygen consumption was most likely covering the cellular ATP requirements, resulting from the mitochondrial proton leak. These OCR values, however, did not reach a statistical significance when compared to untreated control cells (Fig. 2, panel I). Moreover, exposition to PM did not affect monocyte maximal respiration (Fig. 2 Ie) and spare respiratory capacity (Fig. 2 If), while it increased the OCR of the non-mitochondrial respiration phase (Fig. 2 Ig), suggesting a damage of the mitochondrial respiratory chain.

### PM treatment activates Caspase-3-dependent pyroptotic pathway

The formation of ROS, disruption of mitochondrial membrane potential and alterations in mitochondrial respiration may lead to cell death. Therefore, to better characterize the mechanism leading to death of monocytes exposed to PM, activation of Apaf-1, Caspase-9, Caspase-3 and GSDME was determined.

Apaf-1 expression was investigated by Western blot analysis. Obtained results revealed that a 15 min treatment with NIST or LAP meaningfully increased the expression of Apaf-1 in the cytoplasm of monocytes (Fig. 2 IIa and Supplementary Fig. 3a). Interestingly, similar pattern was observed after cell stimulation with TBHP, but not with LPS, thus these results provided evidence that inorganic components of PM may play a role in the activation of Caspase-3 dependent pyroptotic pathway.

Caspase-9 activation was detected by flow cytometry and presented as frequency of FITC-LEHD-FMK (green fluorescence) positive cells. Results revealed the increased activation of Caspase-9 after a 15 min exposure of monocyte to PM (Fig. 2 IIb and IIc). Activation of Caspase-9 was also observed after a 2 h PM treatment, however, the percentage of Caspase-9 positive cells was lower than after 15 min (Supplementary Fig. 2 IIa). Pre-incubation of cells with Mito-TEMPO prior to PM exposure effectively inhibited activation of Caspase-9 in monocytes exposed to PM, proving that the activity of Caspase-9 is ROS-dependent (Fig. 2 IIb and Supplementary Fig. 2 IIa).

In the case of Caspase-3, results indicated the increase of cells with active enzyme after a 15 min exposure to NIST or LAP (Fig. 2 IIb and IId). Moreover, the level of active Caspase-3 positive monocytes remained constant after 2 and 4 h of the exposure to NIST or LAP, but at a lower level. The re-challenge with the same dose of PM for additional 15 min and 2 h only slightly increased the percentage of Caspase-3 positive cells (Supplementary Fig. 2 IIb and IIc).

Cleavage of GSDME is the last component of the Caspase-3-dependent pyroptotic pathway. This was evaluated by Western blot. Obtained results showed that NIST and LAP treatment caused cleavage of GSDME induced by Caspase-3, thus converting apoptotic signals to pyroptosis (Fig. 2 IIe and Supplementary Fig. 3b and c). This effect was more pronounced for LAP than for NIST treatment, proving the role of inorganic fraction of air pollution in the activation of Caspase-3-dependent pyroptotic pathway.

### PM treatment activates the canonical inflammasome pathway

The canonical inflammasome pathway involves activation of inflammasomes, including NLRP3, and Caspase-1, which cleaves GSDMD to the N-terminal domain, initiating pyroptosis and IL-1β secretion.

The data confirmed that short NIST or LAP treatment significantly boosted the expression of NLRP3 in monocytes (Fig. 2 IIIa). This effect was most prominent for NIST particles, proving the involvement of organic components (including endotoxin (LPS) content in PM [57]) in the activation of inflammasomes, including NLRP3 (Fig. 2 IIIa and Supplementary Fig. 3d). Moreover, cell pre-incubation with Mito-TEMPO prior to PM exposure inhibited NLRP3 expression in monocytes exposed to PM, indicating the additional role of ROS in the inflammasome activation (Fig. 2 IIIb and Supplementary Fig. 3e). As the use of MCC950 – the specific inhibitor of NLRP3 activation did not completely block all cell death (Fig. 2 IIIc, IIId and Supplementary Fig. 3f), we cannot exclude the role of other inflammasomes, e.g., NLRP6, NLRC4 or NLRP1b in this phenomenon.

Caspase-1 activity was detected by flow cytometry and presented as a frequency of FAM-YVAD-FMK (green fluorescence) positive cells. Results showed an increase in the level of Caspase-1 positive cells after a 15 min exposure to NIST or LAP (Fig. 2 IIIe and IIIf). Similar pattern was observed for both types of PM. Additionally, pre-incubation of cells with Ac-yvad-cmk, prior to PM, effectively inhibited Caspase-1 activation in monocytes exposed to PM (Fig. 2 IIIe). An increase of Caspase-1 positive cells was also observed after a 2 h NIST treatment, while for LAP particles the frequency of Caspase-1 active monocytes decreased, supporting the role of organic components in Caspase-1 activation (Supplementary Fig. 2 IId). Whereas the longer exposure of monocytes to PM (4 h) resulted in the decrease of Caspase-1 activity, for both types of PM (Supplementary Fig. 2 IIe). Taking into consideration that Ac-yvad-cmk also effectively inhibits ROS production, all these observations suggest that PM play a role in ROS dependent cell death both by direct involvement of inorganic fraction of PM on ROS production and, indirectly, by stimulating ROS formation through Caspase-1, mainly activated by organic fraction of PM. Monocytes re-challenged with the same dose of PM for additional 15 min or 2 h did not show any additional changes in the level of Caspase-1 positive cells (Supplementary Fig. 2 IId and IIe).

GSDMD has been reported to play a key role in the induction of pyroptosis through inflammasomes and Caspase-1 related pathway. Data from Western blot analysis showed that NIST and LAP treatment caused cleavage of GSDMD to GSDMD p30 (Fig. 2 IIIg and Supplementary Fig. 3 g and h). This effect was more evident for NIST than for LAP, highlighting the role of organic components of PM in this signaling path.

The last element of the canonical inflammasome activation pathway is IL-1β, which secretion was determined after monocyte exposure to PM. The level of IL-1β positive cells was evaluated by mouse PE-conjugated anti-human IL-1β monoclonal antibody staining and flow cytometry analysis. The obtained data showed that short exposure to NIST or LAP increased monocyte positive for IL-1β production (Fig. 2 IIIh and IIIi). Pre-incubation of monocytes with Ac-yvad-cmk prior to PM exposure effectively inhibited IL-1β production (Fig. 2 IIIh). The level of IL-1β positive cells was investigated also after the cell exposure to PM for 4 h. The obtained data showed the increase of IL-1β producing monocytes exposed to NIST or LAP (Supplementary Fig. 2 IIIa). Furthermore, to answer whether monocytes, which survived the exposition to PM are able to enhance pro-inflammatory cytokine production, after a 2 h culture with the single dose of NIST or LAP, the cells were re-challenged with the same dose of PM and cultured for additional 2 h. This double PM treatment did not change the level of IL-1β positive cells (Supplementary Fig. 2 IIIa). Additionally, supernatants from monocyte culture were collected after a 4 h PM treatment and the concentration of IL-1β was detected by CBA. Obtained results confirmed that NIST and LAP treatment induces IL-1β production by human monocytes (Supplementary Fig. 2 IIIb).

### PM stimulates pro-inflammatory cytokine production

**Fig. 3:**
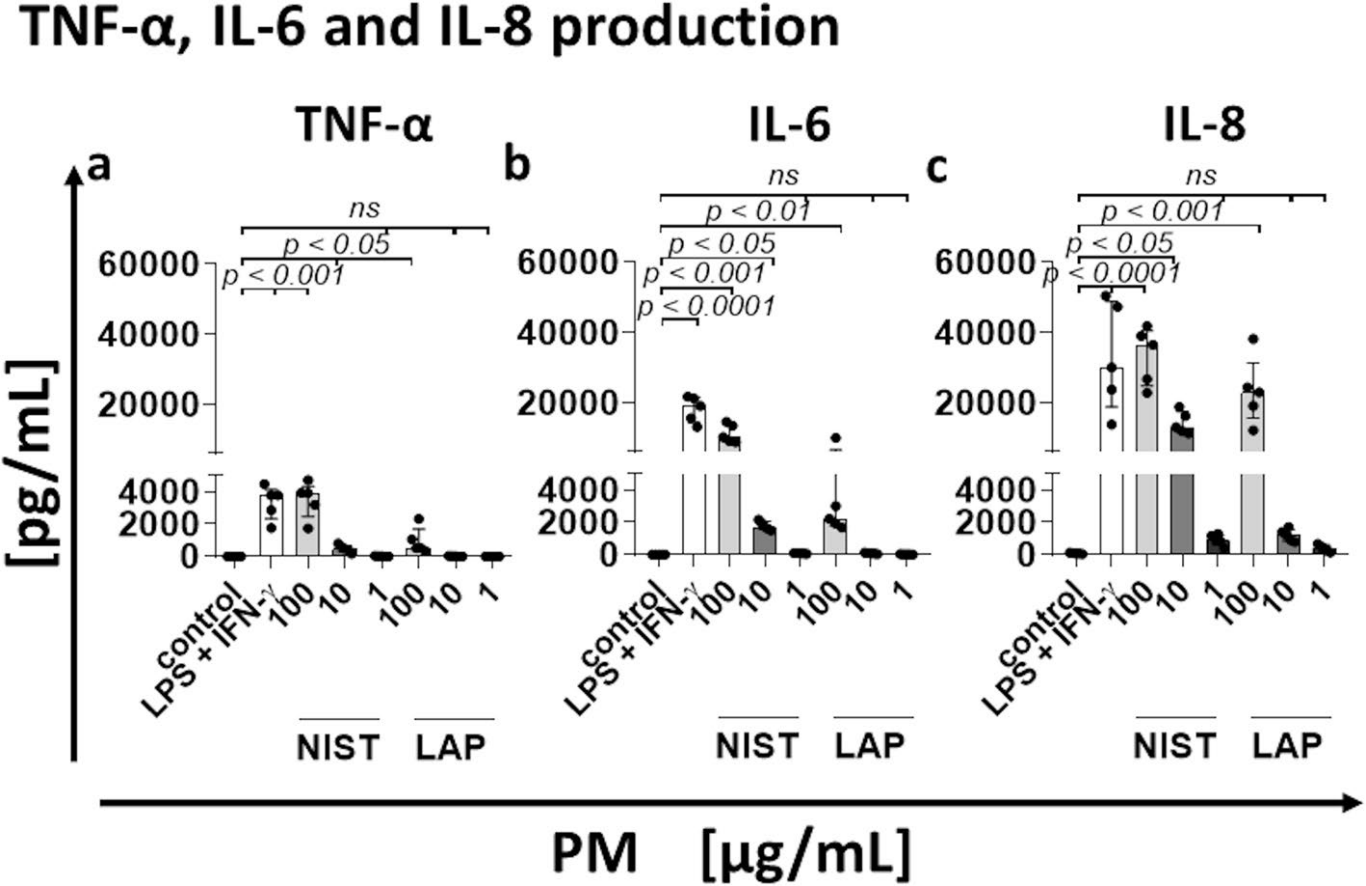
Effect of PM exposure on monocyte cytokine production. Human monocytes were treated with/without NIST or LAP for 4 h of culture. Concentration of TNF-α (**a**), IL-6 (**b**) and IL-8 (**c**) was analyzed in the culture supernatants by Cytokine bead array and flow cytometry analysis. As a positive control, monocytes were stimulated with 400 U/mL of human recombinant IFN-γ and 100 ng/mL of LPS from *Salmonella abortus equi*. Data are presented as median ± interquartile range from 5 independent experiments. Statistically significant differences were estimated at *p* < 0.05, *p* < 0.01, *p* < 0.001, *p* < 0.0001, ns – not significant

In the last set of experiments, the effect of PM treatment on monocyte cytokine production was investigated. Monocytes were incubated for 4 h with or without PM and the concentration of TNF-α, IL-6 and IL-8 in the supernatants was analyzed by CBA. Results showed that monocytes respond to PM treatment by production of TNF-α, IL-6, and IL-8 with the highest concentration recorded for IL-8 (Fig. 3). TNF-α and IL-6 were produced mainly after stimulation with NIST used in a high and medium concentration (100 µg/mL and 10 µg/mL, respectively), while in the case of LAP, it was only noticed for the highest concentration (100 µg/mL), proving the importance of the organic compounds content in PM for cytokine secretion.

## Discussion

A growing list of evidence indicates the correlation between a high concentration of PM in the air and initiation or exacerbation of inflammatory and autoimmune disorders [3, 4]. PM as the component of air pollution may be inhaled, deposited in the respiratory tract where could be phagocytosed by alveolar macrophages or interact with epithelial cells [48, 64]. Smaller PM can be translocated to the blood stream and interact with blood erythrocytes, platelets, and circulating leukocytes [49–53]. In this report, we evaluated the effect of two types of PM, namely NIST and LAP, differing in the content of organic components, on monocyte function and their lifespan. In this aspect, the morphology and cell viability, pro-inflammatory cytokines and ROS production, function of mitochondria, activation of Caspase-1, -3, and -9, and NLPR3 inflammasome were analyzed.

This report showed that PM treatment reduced capacity of human monocytes to induce antigen-driven T cell proliferation. This was evaluated using PPD, the antigen which requires processing by antigen-presenting cells (APCs) [65]. Although we have no formal proof, it’s very likely that PM exposure affects antigen-presenting capacity of monocytes, thus reducing T cell proliferation in response to recall antigen, namely PPD. So far, a decreased antigen presenting capacity of human monocytes was observed in many bacterial and virus infections, including Covid-19 [66–70]. Chaudhuri et al., documented binding and phagocytosis of diesel exhaust particles by human monocytes, causing cell death [71]. In another study Croft et al., observed that increased concentration of black carbon was associated with downregulation of pathways involved in antigen presentation [72]. Our data also indicate that PM can be internalized by monocytes, inducing cell death. Both cytochalasin D treatment and monocyte incubation with PM on ice could significantly reduce the level of Annexin-V positive cells, supporting the role of phagocytosis in PM internalization and induction of monocyte pyroptosis (Supplementary Fig. 1a).

A reduced T-cell proliferation response to PPD prompted us to analyze morphology and viability of monocytes after their exposure to PM. Aggregation of monocytes around the PM agglomerates and their interactions affected cell morphology already after a short time exposure (15 min). Similarly, the aggregation of rat alveolar macrophages exposed to biodiesel particles [60] or human monocytes to diesel exhaust particles was reported [73]. Changes in morphology of monocytes treated with PM corresponded with a drop of their viability. The most pronounced effect was observed for NIST particles, used in the highest concentration (100 µg/mL), what may indicate the role of their organic components [74]. Previous data reported that organic extracts from urban air PM increased apoptosis of human PBMCs [75]. However, in our study decrease of monocyte viability after the exposure to PM was very quick—observed already after 15 min from the treatment. In this context, Green et al., suggested that morphological changes, mitochondrial outer membrane permeabilization and activation of caspases, leading to cell death may be fast and take about 10 min [76]. In keeping, Yu et al., showed fast death through pyroptosis [77]. In our study, a longer exposure of cells to NIST or LAP did not further reduce the cell viability, what may indicate that monocytes react to PM exposure in different manner, most likely due to a different contact level with the particles. In this context it was interesting if the remnant viable monocytes became resistant to PM-induced cell death. This hypothesis was further verified in the experiments where monocytes were re-challenged with the same doses of PM. In this situation we did not observe a decrease of cell viability, measured by Annexin-V binding assay. It is worth mentioning that Annexin-V binding is a common feature shared between pyroptosis and apoptosis. During apoptosis, phosphatidyl serine translocate to the outer leaflet, resulting in positive cell surface staining with Annexin-V, while during pyroptosis, pores open in the cell membrane, permitting Annexin-V to enter the cell and stain the inner leaflet of the membrane [78].

Our results also indicated that PM increased ROS production by human monocytes approximately 15 min. after the addition of particles to cell cultures. There is evidence that air pollution including PM, ultrafine particles and transition metals increases cellular level of ROS [58, 79–81]. There are three potential sources of cellular ROS, which include NADPH oxidases, uncoupled nitric oxide synthase reaction or electron leak from the mitochondrial respiratory chain [82]. Obtained data clearly indicate that ROS produced by human monocyte exposed to PM are mainly of mitochondria origin. This observation was confirmed by results showing that pre-incubation of monocytes with Mito-TEMPO, an inhibitor of mitochondrial ROS, significantly reduced the PM-mediated ROS production. Moreover, this compound with superoxide and alkyl radical scavenging properties [83, 84], effectively improved cell viability. Evaluating the effects of PM on monocyte ROS production, the composition of NIST and LAP was considered. Our results revealed that LAP particles increased ROS formation more efficiently than NIST did. In this context, the presence of organic components in NIST may contribute to faster cell death of human monocytes thus limiting the cell abilities for ROS production. Moreover, removal of the organic components from NIST (LAP particles) most likely resulted in a significant increase in the concentration of transition metals, intensifying the harmful redox reactions. It is accepted that ROS production may cause a direct mitochondrial damage [77]. Mootha et al., described a correlation between the level of ROS and mitochondrial membrane potential depolarization [26]. A drop of mitochondrial membrane potential after exposure of macrophages to fine particulate matter or monocytes to tobacco smoke has been already documented [85–87], supporting our current observations.

Mitochondrial membrane potential is a key element responsible for maintaining the normal functions of mitochondria. To determine whether the observed increase in ROS production and decrease of mitochondrial membrane potential was paralleled by alterations in mitochondrial respiration, an extracellular flux analysis was performed. The analysis revealed changes in OCR. Interestingly, we found increased OCR after PM treatment of monocytes, what was accompanied by the shift from OXFOS to glycolysis. These findings were confirmed by the results showing an increase of proton leak, which can be a proof of mitochondria damage. It is hypothesized that after the exposure to PM, due to the proton leak which blocks ATP production, monocytes shifted from OXFOS to glycolysis to obtain energy from a rapid metabolism of glucose. Moreover, disruption of OXPHOS and switch to glycolysis may suggest the mechanism of inflammatory response utilizing glycolysis for a fast ATP generation [37, 88].

Usually, the increase in ROS production and alterations of mitochondrial membrane potential contribute to release of cytochrome c, which interacts with Apaf-1, leading to activation of Caspase-9 and Caspase-3 [77]. In this report we showed that NIST or LAP treatment meaningfully increases the expression of Apaf-1 and activates the cascade of Caspases, documented by increased frequency of cells positive for Caspase-9, and for Caspase-3. Moreover, pre-incubation of monocytes with Mito-TEMPO inhibited PM-mediated Caspase-9 activation, which was probably the result of the inhibition of ROS production. Other studies also showed that PM treatment induces activation of Caspase-9 and Caspase-3 [64, 85, 89], what may lead to cell apoptosis. However, activated Caspase-3 may not only induce apoptosis but can also cleave GSDME to N-terminal fragment, which ultimately trigger pyroptosis [27, 90]. This scenario was confirmed in our study, by documenting the cleavage of GSDME.

The organic components in PM may act as pathogen-associated molecular patterns (PAMPs) recognized by Toll-like receptors (TLRs), including Nod-like receptors (NLRs) of human monocytes. In this context, the use of monoclonal antibodies blocking TLR-4 showed a positive impact on monocyte viability, especially with respect to the treatment with NIST particles, further documenting a role of bio-components, including LPS, in this process (Supplementary Fig. 1b). A subset of NLRs named NLRP (multi-molecular complex called inflammasome) can form a structure which can activate Caspase-1 for cleavage of GSDMD to GSDMD-N, which supports the formation of pores in the plasma membrane for IL-1β secretion, leading to pyroptosis [43]. Our data showed that PM increased the expression of NLRP3 in monocytes. Activation of inflammasome NLRP3 after exposure to PM was also observed in other studies [34, 37, 91]. Additionally, our study revealed that PM treatment activated Caspase-1, followed by a cleavage of GSDMD and production of IL-1β. It is worth to mention that this effect was stronger for NIST—the particles with higher organic fraction content. In this context, we suggest that organic components of PM may play a role in the activation of the canonical inflammasome pathway. This was proven using MCC950, the specific inhibitor of NLRP3 activation, which was able to reduce the monocyte Annexin-V binding ability only after the treatment with NIST particles. However, as the effect of MCC950 on the cell viability was only partial, we cannot exclude the role of other inflammasome-related pathways, e.g., NLRP6, NLRC4, NLRP1b. Moreover, a pre-incubation of monocytes with Caspase-1 inhibitor (Ac-yvad-cmk) prior to the exposure to PM revealed that inhibition of Caspase-1 activity also inhibited the ROS production. This observation supports the scenario that ROS production in monocytes after PM treatment is additionally dependent on Caspase-1, which activity can be regulated by the organic fraction of PM.

Our results showed also that longer exposure of monocytes to NIST or LAP did not increase the rate of cell death which may be a result of the protective effect of activation the remnant viable monocytes by released IL-1β and DAMPs. Such monocytes exposed to PM responded with the production of pro-inflammatory cytokines (TNF-α, IL-6, IL-8 and IL-1β). In a similar study, Gawda et al., observed the increase of pro-inflammatory cytokines production (TNF-α, IL-6 and IL-12p40) by murine macrophages after NIST treatment [92]. In another studies, diesel exhaust particles induced TNF-α, IL-1β, IL-6 and IL-8 secretion by human monocytes [74, 93]. The increased level of IL-6, IL-8, and TNF-α produced by human A549 and mouse RAW264.7 cells exposed to PM was also reported [94]. In this context, pattern recognition receptors (PRRs) including TLRs and NLRs may play a crucial role. Several studies indicated that endotoxin may be involved in promoting inflammation induced by PM [95–101]. In our previous report we documented that LPS is a major organic component in NIST, however polymyxin B treatment (inhibitor of LPS activity) did not completely abrogated monocyte proinflammatory cytokine secretion induced by NIST particles, suggesting a role of other organic constituents present in PM samples [57].

## Conclusion

In summary, our report shows that PM exposure has significant impact on monocyte function and induces their death by pyroptosis. This leads to release of pro-inflammatory cytokines by remnant viable monocytes activated by IL-1β and DAMP molecules. Investigating the mechanisms of PM-induced pyroptosis, our results show that PM promoted a shift in monocyte oxygen metabolism from oxidative phosphorylation to glycolysis, enhanced ROS formation followed by drop of mitochondrial membrane potential and further activation of Apaf-1, Caspase-9, and Caspase-3, leading to cleavage of GSDME and initiating the Caspase-3-dependent pyroptotic pathway. At the same time, PM induced the secretion of IL-1β by the canonical inflammasome pathway, cleavage of GSDMD and activation of Caspase-1. Our observations indicate that the composition of PM played a crucial role in the activation of both paths—the inorganic fraction of PM was responsible for the induction of Caspase-3-dependent pyroptotic pathway, while the canonical inflammasome path was activated by the organic components of PM.

### Supplementary Information

**Additional file 1: Supplementary Figure 1.** **Role of phagocytosis and TLR-4 in monocyte death after PM treatment.** (**a**) Monocytes were pre-incubated for 1h with cytochalasin D (0.1 μM; Sigma) or were kept on ice to inhibit phagocytosis; (**b**) or incubated with polyclonal mouse anti-human TLR-4 antibody (5 μg/mL; InvivoGen) to inhibit LPS binding, prior to the PM exposure. Data are presented as ratio of Annexin V positive cells in the treated groups to untreated control (median ± interquartile range from 3 independent experiments). Statistically significant differences were estimated at *p < 0.05, **p < 0.01, ***p < 0.001, ns – not significant.

**Additional file 2: Supplementary Figure 2.** **The effect of PM on monocyte: (I) OCR.** Medium culture, NIST or LAP were used without monocytes to exclude any possible influence on the OCR values (negative control). All measurements were performed in duplicates. **(II) Activation of Caspases-9 (a), -3 (b, c) and -1 (d, e).** The activation of Caspase-9, after 2h exposure to NIST or LAP, was evaluated by Caspase 9 (active) Staining Kit and flow cytometry analysis. Additionally, monocytes were pre-incubated for 1h with 1.5 mM Mito-TEMPO. Caspase-3 activity was detected by staining with PE-conjugated anti-active Caspase-3 monoclonal antibody and flow cytometry analysis after 2 and 4h of the exposure to PM. The cells with active Caspase-1 were evaluated by Caspase-1 (active) Staining Kit and flow cytometry analysis. As a positive control, monocytes were stimulated with 400 U/mL of human recombinant IFN-γ and 100 ng/mL of LPS from *Salmonella abortus equi*. Additionally, cells with Caspase-3 and -1 activity were also determined after 15 min and 2h of culture with the second dose of PM, added after 2h exposition to single dose of PM. Data are presented as a percentage of Caspase-9/-3/-1 positive cells (median ± interquartile range from 4 independent experiments). **(III) IL-1β production.** The IL-1β producing monocytes were evaluated by staining with PE-conjugated mouse anti-human IL-1β monoclonal antibody and flow cytometry analysis. Human monocytes were cultured with single dose or without NIST or LAP for 4h or for 2h with the second dose of PM added after 2h of the exposure to single dose of PM. Data are presented as a percentage of IL-1β positive cells (**a**). Additionally, concentration of IL-1β was determined in the supernatants by CBA and flow cytometry analysis (**b**). As a positive control, monocytes were stimulated with 400 U/mL of human recombinant IFN-γ and 100 ng/mL of LPS from *Salmonella abortus equi.* Data are presented as a median ± interquartile range from 5 independent experiments. **(II and III)** Statistically significant differences were estimated at *p* < 0.05, *p* < 0.01, *p* < 0.001, *p* < 0.0001, ns – not significant.

**Additional file 3: Supplementary Figure 3.** **The effect of PM on the expression of Apaf-1, GSDME, NLRP3 and GSDMD.** Activation of Apaf-1 (**a**), GSDME (**b**, **c**), NLRP3 (d, **e**, **f**) and GSDMD (**g,** **h**) after 15 min of monocyte exposure to NIST or LAP (100 µg/mL) was evaluated by Western blot and densitometric analysis. GAPDH was used to normalized protein expression. Data are presented as a median ± interquartile range from 3 independent experiments. Statistically significant differences were estimated at *p* < 0.05, *p* < 0.01, ns – not significant.

## Data Availability

The datasets used and/or analyzed during the current study are available from the corresponding author on reasonable request.

## Abbreviations

AIM2: Absent in melanoma 2
Apaf-1: Apoptotic protease activating factor 1
ASC: Apoptosis-associated speck-like protein containing a CARD
CCCP: Carbonyl cyanide m-chlorophenylhydrazone: DAMPs, danger-associated molecular patterns
DMEM: Dulbecco's Modification of Eagle's Medium
FCCP: Carbonyl cyanide-4-(trifluoromethoxy)phenylhydrazone
GSDMD: Gasdermin D
GSDME: Gasdermin E
HD: Healthy donors
LAP: SRM 1648a with the organic fraction removed
LPS: Lipopolysaccharide
MCC950: N-((1,2,3,5,6,7-heksahydro-s-indacen-4-ylcarbamoyl)-4-(2-hydroxy-2-propanyl)-2-furansulfonamide)
Mito-TEMPO: (2-(2,2,6,6-Tetramethylpiperidin-1-oxyl-4-ylamino)-2-oxoethyl) triphenylphosphonium chloride
ΔΨMMP: Changes of the mitochondrial membrane potential
NIST: SRM 1648a, standard urban particulate matter from the US National Institute for Standards and Technology
NLRP: Nucleotide-binding oligomerization domain-like receptor pyrin domain-containing
NLRP3: Nucleotide-binding oligomerization domain-like receptor pyrin domain containing 3
NLRs: Node-like receptors
OCR: Oxygen consumption rate
OXPHOS: Oxidative phosphorylation
PAMPs: Pathogen-associated molecular patterns
PBMCs: Peripheral blood mononuclear cells
PM: Particulate matter
PPD: Purified protein derivative of tuberculin
PRRs: Pattern recognition receptors
ROS: Reactive oxygen species
RPMI: Roswell Park Memorial Institute
SRM 1648a: Standard reference material
TBHP: Tert-butyl hydroperoxide
TLRs: Toll-like receptors
TM: Transition metals
