# Supplementary figures and images for: Air pollution induces pyroptosis of human monocytes through activation of inflammasomes and Caspase-3-dependent pathways
Source: J Inflamm (Lond). 2023 Aug 10;20:26. doi: 10.1186/s12950-023-00353-y (PMC10416410; doi:10.1186/s12950-023-00353-y)

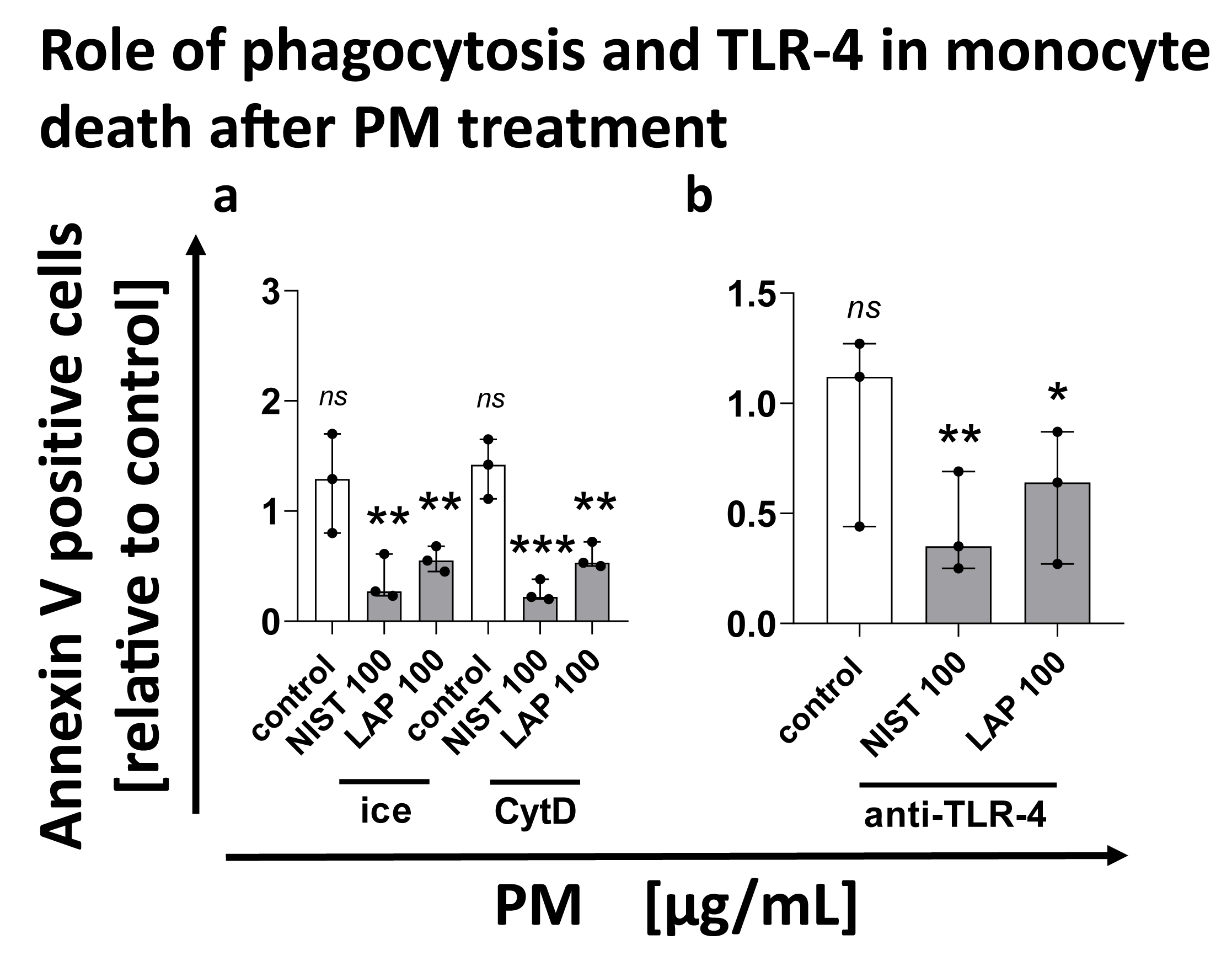

Supplement: Supplementary file 1 — Additional file 1: Supplementary Figure 1. Role of phagocytosis and TLR-4 in monocyte death after PM treatment. (a) Monocytes were pre-incubated for 1h with cytochalasin D (0.1 μM; Sigma) or were kept on ice to inhibit phagocytosis; (b) or incubated with polyclonal mouse anti-human TLR-4 antibody (5 μg/mL; InvivoGen) to inhibit LPS binding, prior to the PM exposure. Data are presented as ratio of Annexin V positive cells in the treated groups to untreated control (median ± interquartile range from 3 independent experiments). Statistically significant differences were estimated at *p < 0.05, **p < 0.01, ***p < 0.001, ns – not significant. [file 12950_2023_353_MOESM1_ESM.tif]

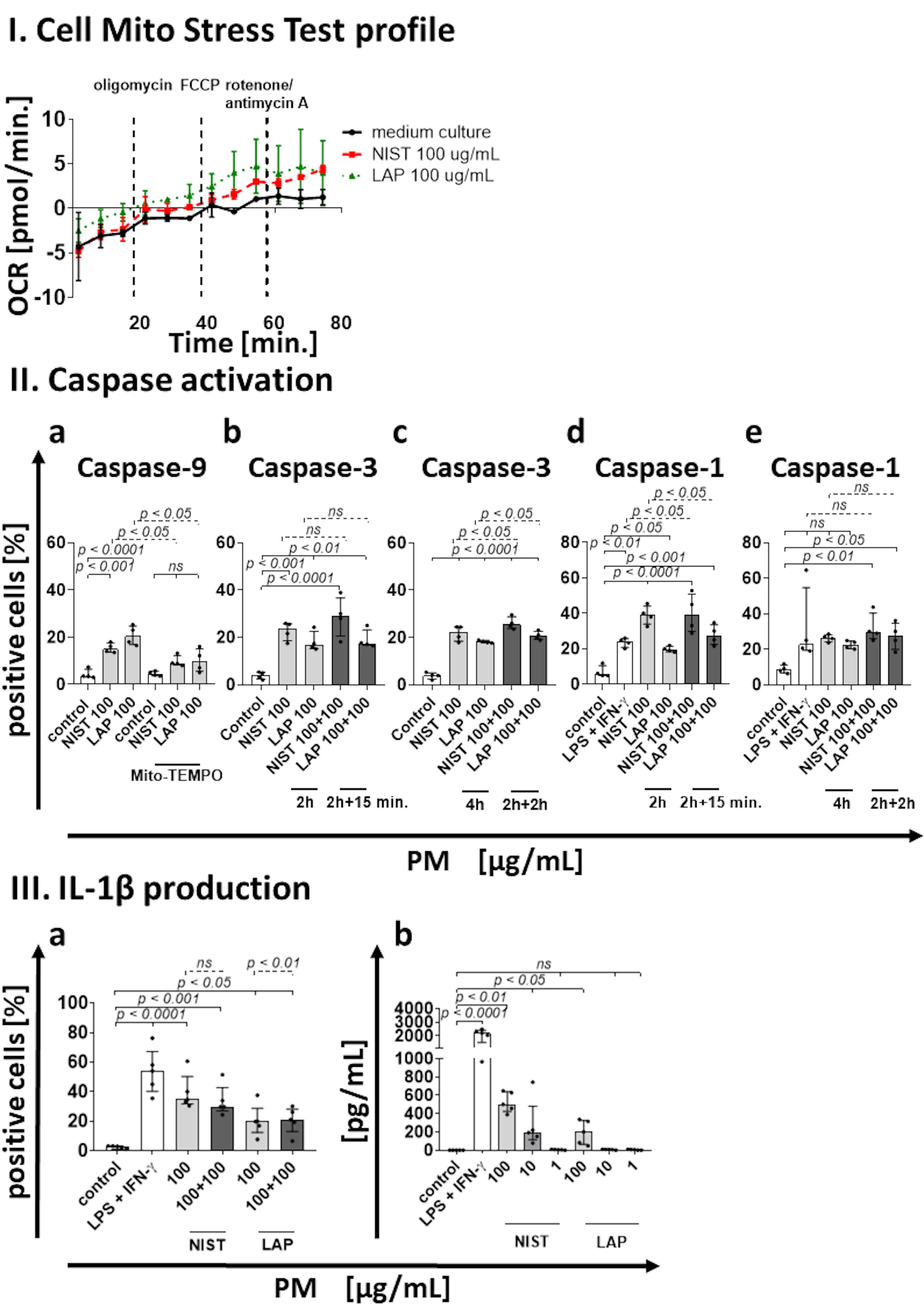

Supplement: Supplementary file 2 — Additional file 2: Supplementary Figure 2. The effect of PM on monocyte: (I) OCR. Medium culture, NIST or LAP were used without monocytes to exclude any possible influence on the OCR values (negative control). All measurements were performed in duplicates. (II) Activation of Caspases-9 (a), -3 (b, c) and -1 (d, e). The activation of Caspase-9, after 2h exposure to NIST or LAP, was evaluated by Caspase 9 (active) Staining Kit and flow cytometry analysis. Additionally, monocytes were pre-incubated for 1h with 1.5 mM Mito-TEMPO. Caspase-3 activity was detected by staining with PE-conjugated anti-active Caspase-3 monoclonal antibody and flow cytometry analysis after 2 and 4h of the exposure to PM. The cells with active Caspase-1 were evaluated by Caspase-1 (active) Staining Kit and flow cytometry analysis. As a positive control, monocytes were stimulated with 400 U/mL of human recombinant IFN-γ and 100 ng/mL of LPS from Salmonella abortus equi. Additionally, cells with Caspase-3 and -1 activity were also determined after 15 min and 2h of culture with the second dose of PM, added after 2h exposition to single dose of PM. Data are presented as a percentage of Caspase-9/-3/-1 positive cells (median ± interquartile range from 4 independent experiments). (III) IL-1β production. The IL-1β producing monocytes were evaluated by staining with PE-conjugated mouse anti-human IL-1β monoclonal antibody and flow cytometry analysis. Human monocytes were cultured with single dose or without NIST or LAP for 4h or for 2h with the second dose of PM added after 2h of the exposure to single dose of PM. Data are presented as a percentage of IL-1β positive cells (a). Additionally, concentration of IL-1β was determined in the supernatants by CBA and flow cytometry analysis (b). As a positive control, monocytes were stimulated with 400 U/mL of human recombinant IFN-γ and 100 ng/mL of LPS from Salmonella abortus equi. Data are presented as a median ± interquartile range from 5 independent experi [file 12950_2023_353_MOESM2_ESM.tif]

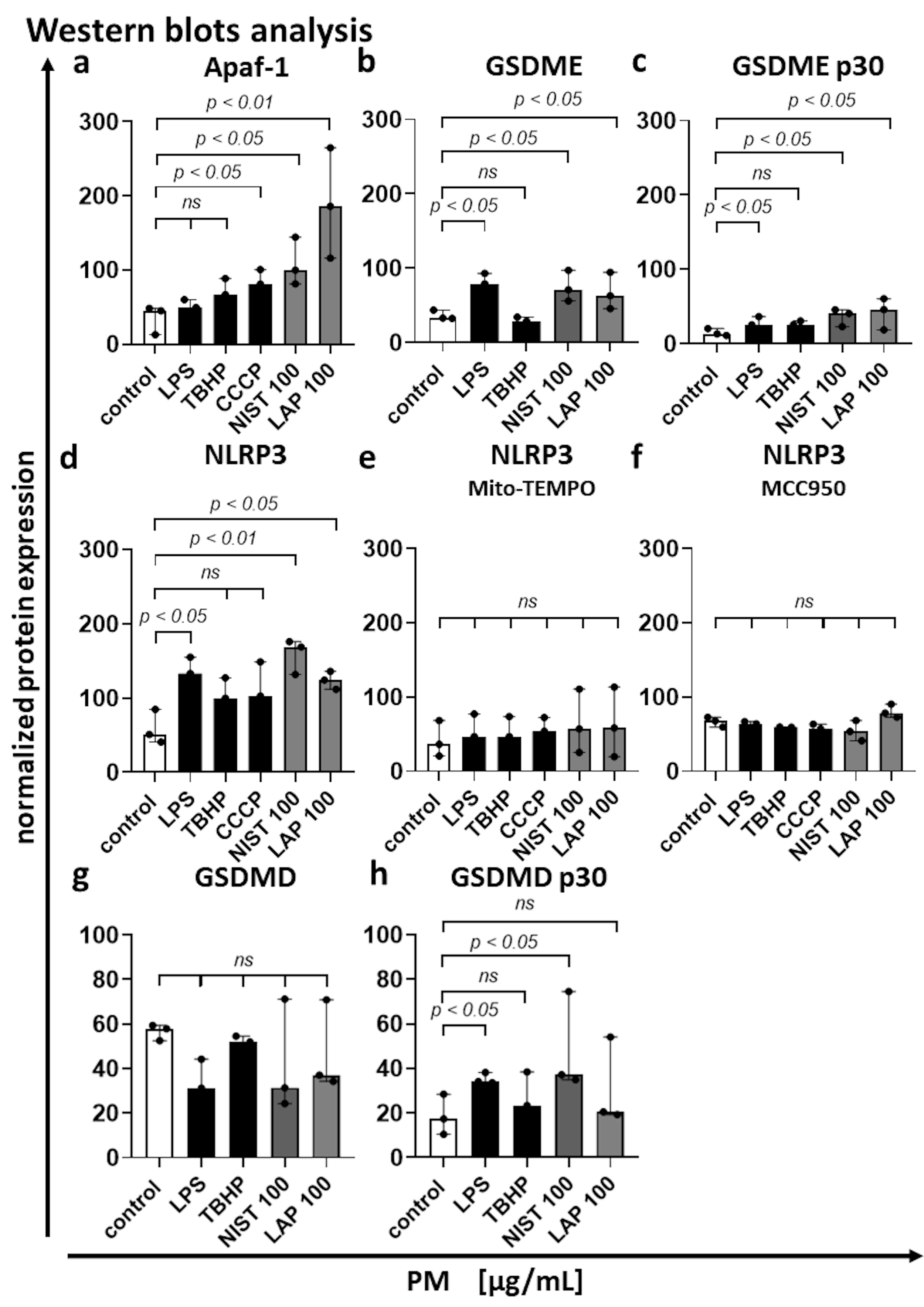

Supplement: Supplementary file 3 — Additional file 3: Supplementary Figure 3. The effect of PM on the expression of Apaf-1, GSDME, NLRP3 and GSDMD. Activation of Apaf-1 (a), GSDME (b, c), NLRP3 (d, e, f) and GSDMD (g, h) after 15 min of monocyte exposure to NIST or LAP (100 µg/mL) was evaluated by Western blot and densitometric analysis. GAPDH was used to normalized protein expression. Data are presented as a median ± interquartile range from 3 independent experiments. Statistically significant differences were estimated at p < 0.05, p < 0.01, ns – not significant. [file 12950_2023_353_MOESM3_ESM.tif]
